# Pneumocystis Jirovecii Pneumonia in Newly Diagnosed HIV Infection: A Challenging Case Report

**DOI:** 10.5146/tjpath.2020.01480

**Published:** 2020-09-15

**Authors:** Selin Kestel Kayık, Elif Acar, Leyla Memiş

**Affiliations:** Department of Pathology, Gazi University, School of Medicine, Ankara, Turkey

**Keywords:** Acquired Immunodeficiency Syndrome, Pneumocystis pneumonia, HIV

## Abstract

*Pneumocystis jirovecii* is a potentially life-threatening opportunistic pathogen particularly affecting the lungs, mainly in immunosuppressed individuals and HIV-infected patients with a low CD4 cell count. A 50-year-old man presented with a 1-week history of pleuritic chest pain and fever. He was also hypoxic with oxygen saturation of 86% on room air. Detailed clinical history revealed that he had fatigue, dyspnea, night sweats, generalized bone pain and a loss of about 10 kg in weight over the past six months without intention. Chest imaging showed diffuse bilateral infiltrates. Diagnostic bronchoscopy was performed. Transbronchial biopsy and bronchoalveolar lavage were received simultaneously. The presence of *P. jirovecii* was suspected in hematoxylin-eosin-stained slides, and Gomori’s methenamine silver stain was used to confirm the diagnosis. A blood test revealed dyslipidemia, hypothyroidism, increased plasma levels of the gonadotropins and positive HIV antibodies with a CD4+ cell count of 48/μL. CMV co-infection was found with CMV viral load of 6738 copies/ml in plasma. Herein, we present a case with *Pneumocystis jirovecii* pneumonia (PCP) that led to a new diagnosis of Human immonudeficiency virus. As in our case, diagnosis of disease through the pathological examination of tissues (biopsy samples) or bodily fluids could lead to the recognition of an unrevealed HIV-infection.

## INTRODUCTION

The incidence of AIDS and patients infected with HIV have greatly increased in number since the 1980s. *Pneumocystis* pneumonia quickly became one of the main AIDS-defining diseases in the late 1980s (1). There are currently 37.9 million people living with HIV. 1.7 million people were newly infected with HIV in 2018 and 770,000 people died of AIDS-related illnesses (2).

*Pneumocystis* was first recognized in an animal that was infected with *Trypanosoma cruzi*. It was therefore thought that *Pneumocystis* was a form in the life cycle of *T. cruzi*. The one who first recognized* Pneumocystis* in the lungs of an experimentally infected rat with *T. Lewisi *was Antonio Carinii. This new species of *Pneumocystis* was named *P. carinii* and caused infection in rat lungs. *Pneumocystis* was also described as the causative agent of interstitial plasma cell pneumonia in the lungs of premature debilitated babies in nurseries and foundling hospitals in Central Europe by Van der Meer and Brug in 1942 (3). However, the most convincing evidence was established by Vanek and Jirovec in 1952. Vanek and Jirovec identified *Pneumocystis* as the causative agent of this disease from autopsies of 16 cases (4). Firstly *Pneumocystis* was accepted as protozoa, but then it was proved by DNA analysis that *Pneumocystis* is a close relative of fungi rather than protozoa (5). The *Pneumocystis* organisms which infect humans are named *P. jirovecii*. Impairment of cell-mediated immunity is especially important for the development of PCP. This relationship is proved by the co-occurrence of PCP in patients with AIDS. Other common populations that are under the risk of developing PCP are organ transplant patients, cancer patients, and patients on chemotherapeutic agents and immunosuppressant drugs such as steroids, cytotoxic agents and anti-tumor necrosis factor drugs used for rheumatologic diseases (6).

We describe a case with *Pneumocystis* pneumonia and newly diagnosed Human Immunodeficiency Virus in a patient with nonspecific symptoms. Besides PCP, a detailed examination revealed endocrine abnormalities with an untreated advanced HIV infection at first admission.

## CASE REPORT

**Figure 1:**
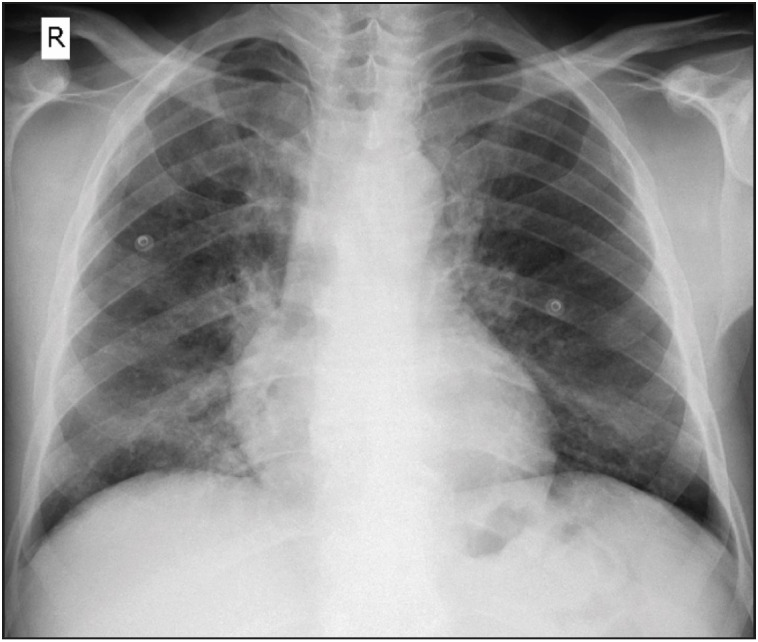
Bilateral diffuse symmetric reticular interstitial infiltrates.

**Figure 2:**
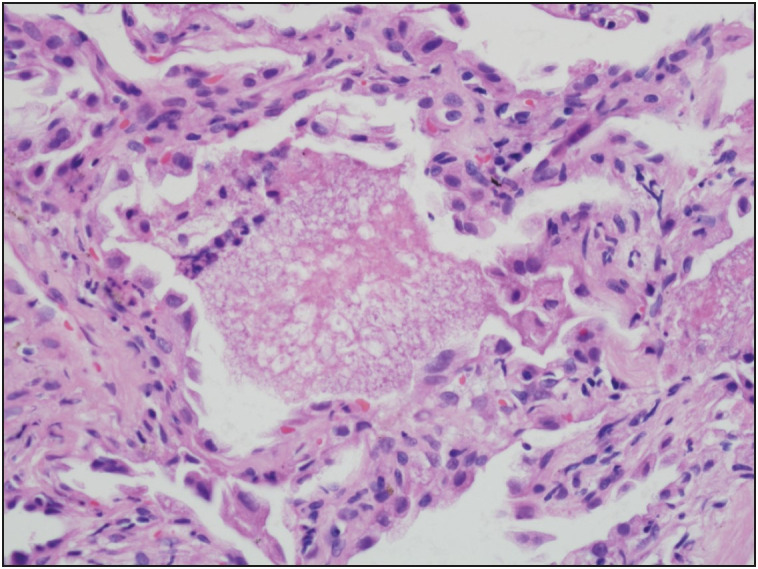
An alveolus with the foamy/bubbly eosinophilic exudates and a few mononuclear cells. The alveolar septa have a few inflammatory cells and collagen and are lined by hyperplastic type II pneumocytes (H&E; x600).

**Figure 3:**
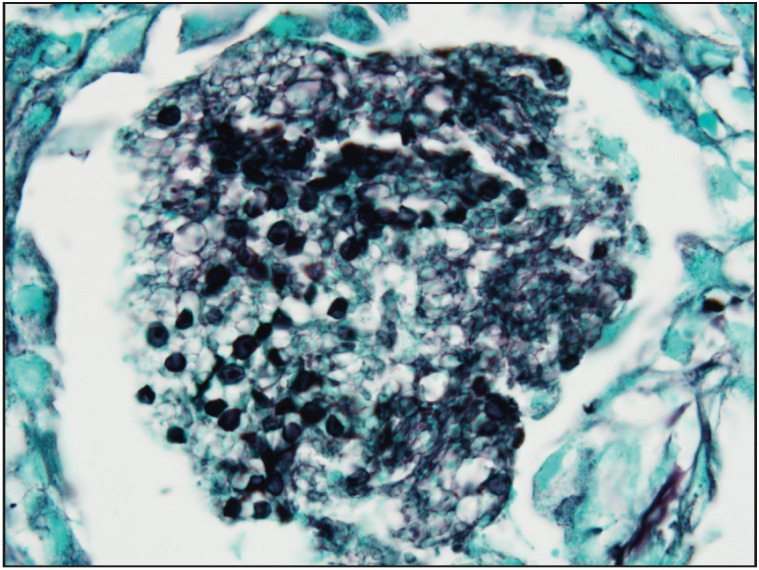
Gomori Methenamine-Silver (GMS) stain of transbronchial biopsy shows Pneumocystis jirovecii cysts with capsular dots. Note that the position of the dots varies in each cyst (GMS; x1000).

**Figure 4:**
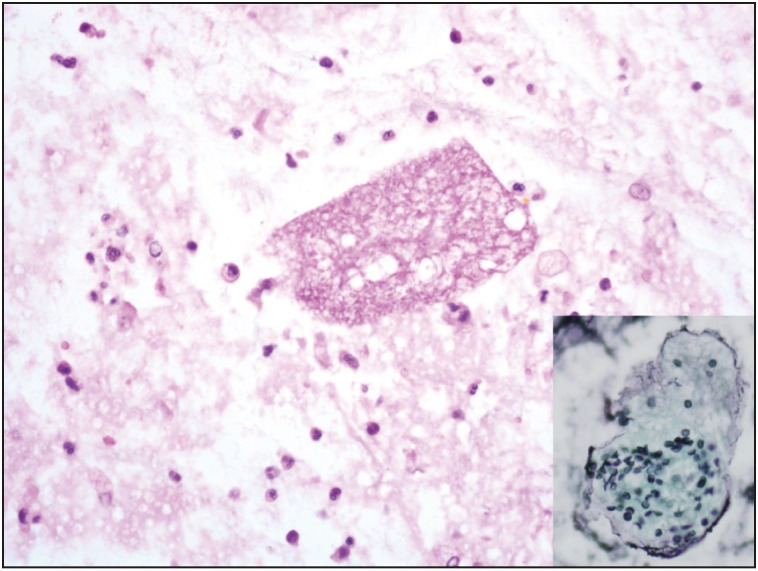
The foamy/bubbly eosinophilic exudates and a few mononuclear cells in the bronhoalveolar lavage fluid cell block (H&E; x600). Inset image: GMS stain decorates the crescentic cyst walls within the foamy material (GMS; x1000).

A 50-year-old man presented with a 1-week history of pleuritic chest pain and fever. His body temperature was 38.4 °C. He was also hypoxic with oxygen saturation of 86% on room air, requiring 3-4 L of oxygen per nasal cannula. The detailed clinical history revealed that he had fatigue, dyspnea, night sweats, generalized bone pain and an unintentional loss of about 10 kg in weight over the past six months. He has no known disease or surgical operation history. He did not use any medication. Physical examination was noteworthy for basal rales bilaterally. Laboratory examinations showed an elevated ESR 102 mm/hr (normal reference 1-15 mm/hr), CRP 79.6 mg/L (normal reference 0-5 mg/L), ProBNP 171 pg/mL (normal reference 0-110 pg/mL), TSH 10,604 mIU/mL (normal reference 0.38-5.33 mIU/mL), antithyroglobulin antibodies 5.7 IU/mL (normal reference 0-4 IU/mL), FSH 27.14 mIU/mL (normal reference 1.27-19.26 mIU/mL), LH 13.47 mIU/mL (normal reference 1.24-8.62 mIU/mL), LDL 137.9 mg/dL (normal reference 60-130 mg/dL), total cholesterol 216.6 mg/dL (normal reference 0-200 mg/dL), HDL 30.7 mg/dL (normal reference 40-60 mg/dL), triglycerides 240 mg/dL (normal reference 0-200 mg/dL) and low levels of Hemoglobin at 10.8 g/dL (normal reference 13.5-17.5 g/dL). WBC count was 8.06×109/L (normal reference 4.5 to 11.0 ×109/L). A chest X-ray showed diffuse bilateral infiltrates (Figure 1). Firstly, the patient was started empirically with ceftriaxone and clarithromycin for a presumed diagnosis of infection, and prednisone for respiratory distress. Diagnostic bronchoscopy with bronchoalveolar lavage and transbronchial biopsy was done for etiological investigation. Light microscopic examination of the transbronchial biopsy showed foamy, pale eosinophilic, amorphous material in the alveolar spaces (Figure 2). Next to these areas, the thickness of the alveolar septum was increased with lymphoplasmacytic inflammatory cell infiltration. A predominance of type 2 pneumocytes was noticed in the alveolar walls (Figure 2). The differential diagnostic list included pulmonary alveolar proteinosis, alveolar edema, and* Pneumocystis jirovecii *pneumonia. Alveolar proteinosis was excluded since there were no cholesterol clefts and PAS-positivity in the intra-alveolar material. With Gomori’s methenamine silver nitrate method, cysts of *Pneumocystis jirovecii* stained brownish-black with capsular dots (Figure 3, Figure 4).

HIV antibody testing was performed, and the patient tested positive. The CD4 count was 48 cells/μm (normal reference range 500-1500 cells/μm) and viral load was very high (1 260 000 copies). CMV co-infection was found with CMV viral load of 6738 copies/ml in plasma.

The patient has been followed up for two years and had two episodes of CMV pneumonia plus one of *Pneumocystis jirovecii* pneumonia

## DISCUSSION

HIV infection is closely related to opportunistic infections and tumors that are mostly seen in immunosuppressed individuals. In the acute phase of HIV infection, CD4+ T cells in mucosal tissue are infected (7). Mucosal infection is followed by the dissemination of the virus and the development of antiviral humoral and cell-mediated host immune responses. These responses are evidenced by seroconversion that is usually detectable within 3 to 7 weeks of presumed exposure. In this stage, 40% to 90% of people who acquire a primary infection develop acute retroviral syndrome. Clinically it is a self-limited acute illness with nonspecific symptoms such as sore throat, myalgias, fever, weight loss, and fatigue, resembling a flu-like illness. These clinical findings resolve spontaneously in 2 to 4 weeks. During this period, major transmission of infection can happen even before the antibody response has appeared (8). If individuals have tissue biopsies in this period, it is possible to detect HIV by immunochemistry (9). In the chronic phase of the HIV infection, patients are either asymptomatic or develop minor opportunistic infections such as oral candidiasis, vaginal candidiasis or herpes zoster. Finally, when the host defense loses the battle, a dramatic increase in the plasma viral load and life-threatening severe clinical HIV disease appears, originally termed as acquired immune deficiency syndrome (AIDS) (10).

*Pneumocystis* is an extracellular organism that has a major predilection for the lung and mostly inhabits alveolar spaces (11). *P. jirovecii* is still the most common cause of lethal pneumonia in patients with HIV infection. Although it is not certain, the transmission of *Pneumocystis* from host to host is assumed to occur via aerosolized particles (12). *Pneumocystis* organisms have at least 2 predominant life cycle forms. The trophic form measures between 1-4 µm, is relatively pleomorphic in shape, is found in clusters, is surrounded by a plasma membrane, and has no rigid cell wall. For detection of the trophozoite form, a variety of Romanowsky stains (Giemsa, Wright, Diff-Quick), and Gram and methylene blue stains can only be applied to imprint smears and cytology specimens (13). The cyst form is 5 to 7 µm in diameter, appearing as thick-walled spherules. When collapsed, the cyst form looks like a cup or crescent shape, contains up to eight intracystic bodies. The cyst wall-stains can be used for tissue sections and include Gomori’s (Grocott) methenamine silver (GMS) and its rapid variants, toluidine blue O, and Gram-Weigert methods. GMS is mostly preferred for daily routine diagnostic work by pathologists. We also used GMS to observe *P. jirovecii* in our case (Figure 3, Figure 4). For diagnostic purposes, various methods can be used. The output of these diagnostic specimens according to Gigliotti & Limper & Wright, 2014 is approximately as follows; induced sputum 20-40%, tracheal aspirate 50-60%, bronchoalveolar lavage 75-95%, transbronchial biopsy 75-95%, and open lung biopsy 90-100% (3). In immunocompromised patients, especially those with impaired CD4+ T cell function, pneumocystis organisms begin to proliferate in alveolar spaces and cause fatal infection if untreated. The key inflammatory cells which evoke inflammation in *Pneumocystis* infection include CD4+ T cells, alveolar macrophages, and neutrophils. Type 1 pneumocyte degeneration, hyperplasia of type 2 pneumocytes, and impairment of the alveolar-capillary barrier are observed during advanced infection. As a result, the alveolar gas exchange is disrupted. Surfactant dysfunction is also observed in *P. jirovecii* pneumonia cases.

The typical histopathological pattern in PCP in the individual with AIDS includes eosinophilic, foamy exudate in the alveolar lumen which contains cell debris and the microorganisms (Figure 2). The alveolar exudate may be focal or diffuse. There is a retraction artifact between alveolar exudate and the alveolar septa in formalin-fixed tissue (Figure 2). This feature is also characteristic of *Pneumocystis jirovecii* pneumonia.

Pulmonary alveolar proteinosis (PAP) and pulmonary edema also cause a similar foamy exudate in the H & E stained sections. In PAP, there are cholesterol clefts and lipid-containing macrophages in alveolar spaces. In our case, there was a typical honeycomb appearance of alveolar exudate which is typical for *P. jirovecii* pneumonia. At higher magnification, the microorganisms appear as tiny basophilic dots within the spaces of the frothy exudate. In GMS-stained sections, organisms appear as small (6-8 μm), oval to round or crescent-shaped. The cyst walls are black and characteristically there is a focal, darkly staining area of capsular thickening.

*P. jirovecii *does not bud and this feature can be used to distinguish between this organism and fungi, such as small variants of *Blastomyces dermatitidis*, *Candida glabrata*, capsule deficient *Cryptococcus* species and *Histoplasma capsulatum* (14).

There are commercially available antibodies to *P. jirovecii* in routinely processed cytology and biopsy specimens. They can identify both the cyst and the trophozoite forms of *P. jirovecii* in clinical specimens (15).

There are also molecular techniques such as real-time PCR for the detection of the organism. One should be careful during the assessment of the real-time PCR results as *P. jirovecii* is also found in healthy individuals. The clinical and laboratory results should therefore be considered together to decide whether treatment is necessary or not (16).

*P. jirovecii* cannot be cultivated on cell-free media in the clinical laboratory and the diagnosis of PCP depends mainly on the demonstration of the organism in biopsy or cytology specimens.

Hypoxia is the hallmark of the PCP, but the clinical findings depend on the host immune status. Fever, nonproductive cough, tachypnea, and severe dyspnea may be observed. Cyanosis may be present or may develop rapidly. In acute progressive infection with respiratory failure, diffuse alveolar damage, hyaline membranes and reactive epithelial cell proliferation may be seen. In chronic infection, interstitial and intraluminal fibrosis may be seen (17).

Patients at high risk for PCP, specifically HIV-infected patients with CD4+ T-lymphocyte counts of less than 200 cells/mm3 and all AIDS patients who have already had one or more episodes of PCP receive prophylaxis with TMP-SMX and aerosolized pentamidine (18).

In conclusion, when physicians come across a diagnosis of PCP, HIV infection should be investigated. PCP is a relatively common AIDS-defining infection. As in our case, diagnosis of disease through the pathological examination of tissues (biopsy samples) or bodily fluids could lead to the recognition of an unrevealed HIV infection.

## Conflict of Interest

The authors declare no conflict of interest.

## References

[ref-1] Akgün Kathleen M., Miller Robert F. (2016). Critical Care in Human Immunodeficiency Virus-Infected Patients. Semin Respir Crit Care Med.

[ref-2] (uuuu). The Joint United Nations Programme on HIV/AIDS.

[ref-3] Gigliotti Francis, Limper Andrew H., Wright Terry (2014). Pneumocystis. Cold Spring Harb Perspect Med.

[ref-4] Vanek J., Jirovec O. (1952). [Parasitic pneumonia. Interstitial plasma cell pneumonia of premature, caused by pneumocystis Carinii]. Zentralbl Bakteriol Orig.

[ref-5] Almeida João M. G. C. F., Cissé Ousmane H., Fonseca Álvaro, Pagni Marco, Hauser Philippe M. (2015). Comparative genomics suggests primary homothallism of Pneumocystis species. mBio.

[ref-6] Sabbagh Wissam, Darwich Noor S. (2018). Pneumocystis Jiroveci Pneumonia and Newly Diagnosed Human Immunodeficiency Virus (AIDS) in a 63-Year-Old Woman. Am J Case Rep.

[ref-7] Doitsh Gilad, Galloway Nicole L. K., Geng Xin, Yang Zhiyuan, Monroe Kathryn M., Zepeda Orlando, Hunt Peter W., Hatano Hiroyu, Sowinski Stefanie, Muñoz-Arias Isa, Greene Warner C. (2014). Cell death by pyroptosis drives CD4 T-cell depletion in HIV-1 infection. Nature.

[ref-8] Lucas Sebastian, Nelson Ann Marie (2015). HIV and the spectrum of human disease. J Pathol.

[ref-9] Moonim Mufaddal T., Alarcon Lida, Freeman Janet, Mahadeva Ula, Walt Jon D., Lucas Sebastian B. (2010). Identifying HIV infection in diagnostic histopathology tissue samples--the role of HIV-1 p24 immunohistochemistry in identifying clinically unsuspected HIV infection: a 3-year analysis. Histopathology.

[ref-10] Prevention Centers for Disease Control (uuuu). Revised surveillance case definition for HIV infection-United States, 2014.

[ref-11] Skalski Joseph H., Kottom Theodore J., Limper Andrew H. (2015). Pathobiology of Pneumocystis pneumonia: life cycle, cell wall and cell signal transduction. FEMS Yeast Res.

[ref-12] Alanio Alexandre, Bretagne Stéphane (2017). Pneumocystis jirovecii detection in asymptomatic patients: what does its natural history tell us?. F1000Res.

[ref-13] Ma Liang, Cissé Ousmane H., Kovacs Joseph A. (2018). A Molecular Window into the Biology and Epidemiology of Pneumocystis spp. Clin Microbiol Rev.

[ref-14] Roden Anja C., Schuetz Audrey N. (2017). Histopathology of fungal diseases of the lung. Semin Diagn Pathol.

[ref-15] Lee Jeong Hyeon, Lee Ji Young, Shin Mi Ran, Ahn Hyeong Kee, Kim Chul Whan, Kim Insun (2011). Immunohistochemical identification of pneumocystis jirovecii in liquid-based cytology of bronchoalveolar lavage - Nine cases report. Korean J Pathol.

[ref-16] Töz Seray, Gündüz Cumhur, Tetik Aslı, Taşbakan Meltem, Pullukçu Hüsnü, Bacakoğlu Feza, Taşbakan Mehmet Sezai, Gülen Figen, Ünver Ayşegül, Turgay Nevin (2017). The comparison of microscopy and real time polymerase chain reaction methods for the diagnosis of Pneumocystis Jirovecii pneumonia: evaluation of clinical parameters. Tuberk Toraks.

[ref-17] Suzuki Tetsuya, Shimoda Yukiko, Teruya Katsuji, Gatanaga Hiroyuki, Kikuchi Yoshimi, Oka Shinichi, Watanabe Koji (2019). Case report: new development of fibrosing interstitial lung disease triggered by HIV-related pneumocystis pneumonia. BMC Pulm Med.

[ref-18] Salzer Helmut J. F., Schäfer Guido, Hoenigl Martin, Günther Gunar, Hoffmann Christian, Kalsdorf Barbara, Alanio Alexandre, Lange Christoph (2018). Clinical, Diagnostic, and Treatment Disparities between HIV-Infected and Non-HIV-Infected Immunocompromised Patients with Pneumocystis jirovecii Pneumonia. Respiration.

